# Correction: Bicoid gradient formation and function in the Drosophila pre-syncytial blastoderm

**DOI:** 10.7554/eLife.26811

**Published:** 2017-03-15

**Authors:** Zehra Ali-Murthy, Thomas B Kornberg

Ali-Murthy Z, Kornberg TB. 2016. Bicoid gradient formation and function in the Drosophila pre-syncytial blastoderm. *eLife* **5**:e13222. doi: 10.7554/eLife.13222

Published 17, February 2016

In the published article the *Fixation and de-vitellinization* section of the **Materials and methods**, for Embryo in situ hybridization the wash buffer was listed as containing 1% Triton X-100. The wash buffer should have been described as containing 0.1% Triton X-100, and the relevant sentence should be: Embryos were de-chorionated in 50% bleach, washed with 0.1% Triton X-100 and transferred to a 20 ml vial containing fixative (5 ml heptane, 5 ml phosphate buffered saline (PBS), 50 mM EGTA (pH 8.0), 10.24% formaldehyde (Ultrapure), and shaken for 1.5 hour.

In the *Pre-hybridization* section of the **Materials and methods**, for Embryo in situ hybridization the pre-hybridization solution (HybS) was listed as containing 50% formaldehyde. HybS should have been described as containing 50% formamide. The correct sentence is: Embryos were incubated with rocking for: (1) 10 min at room temperature in 1 ml 1:1 hybridization solution (HybS):PBT (HybS; 50% formamide, 5xSSC, 100 μg/ml salmon sperm DNA, 50 μg/ml heparin, 0.1% Tween 20, 0.3% Triton X-100); (2) 10 min in 1 ml HybS at 55°C; (3) 45 min in 1 ml fresh HybS at 55°C; and (4) overnight in fresh HybS at 55°C.

The article has been corrected accordingly.

